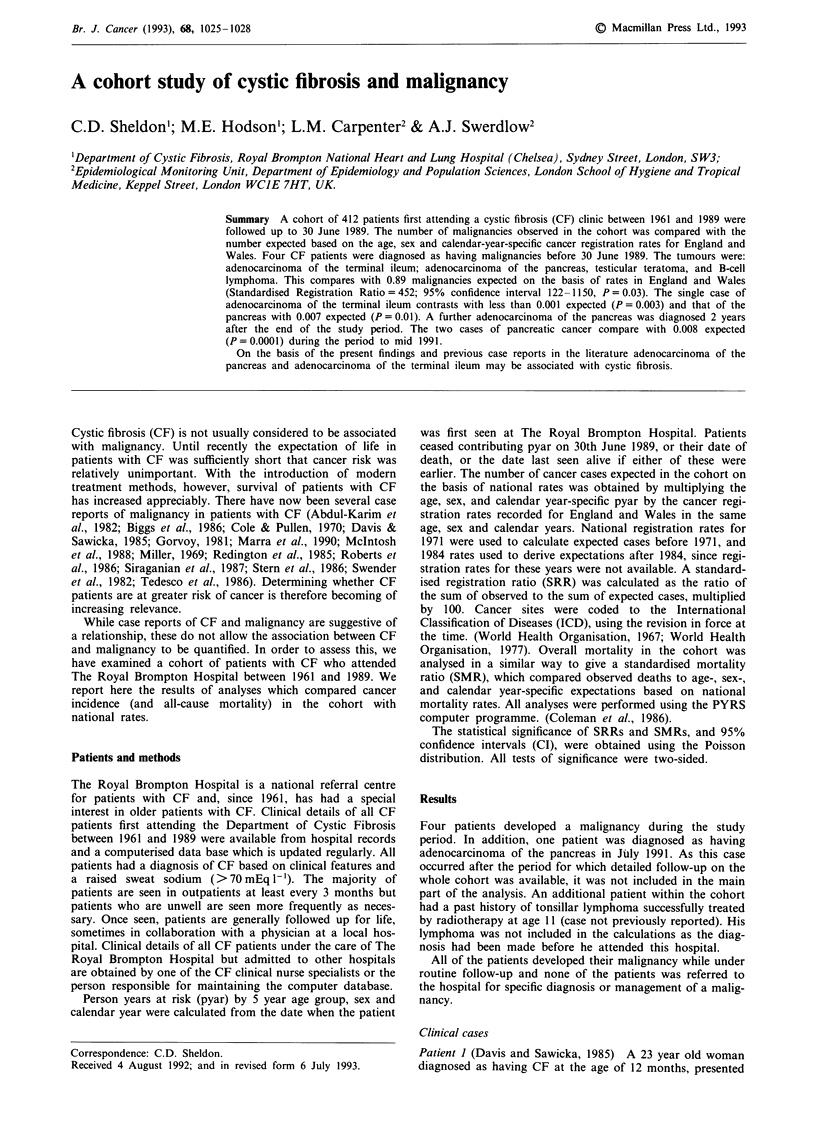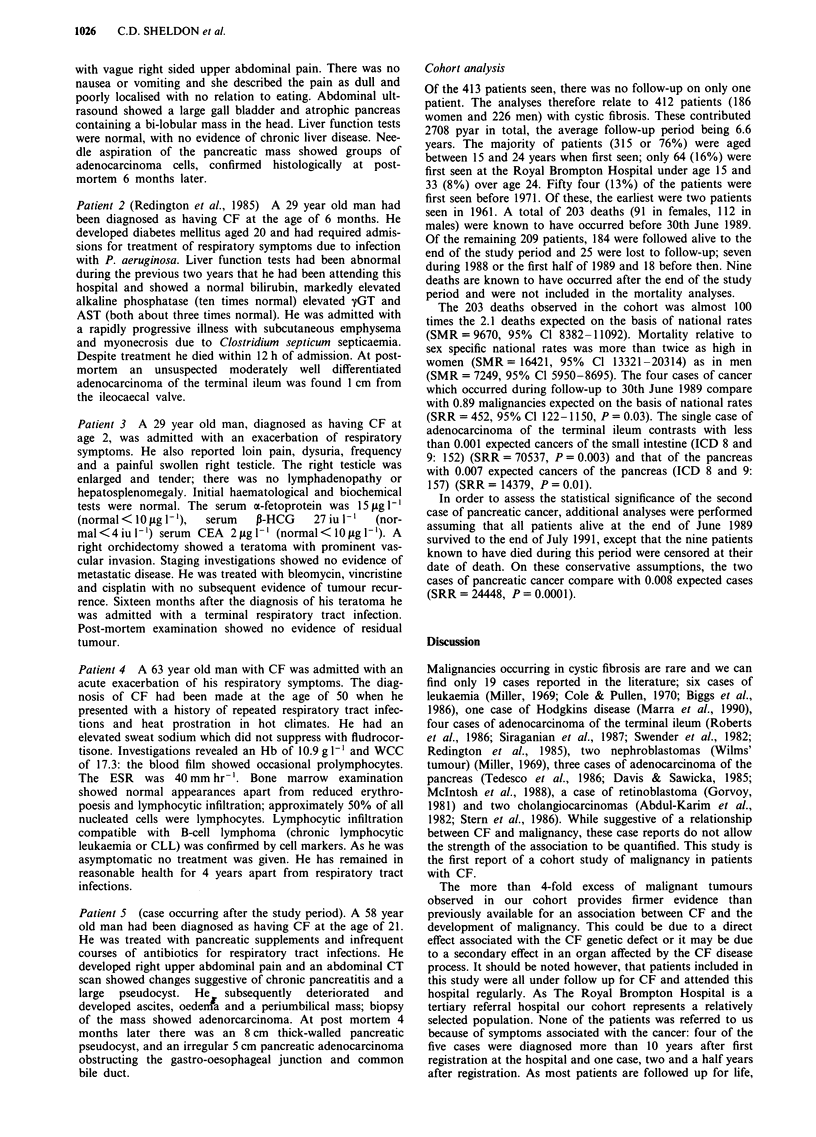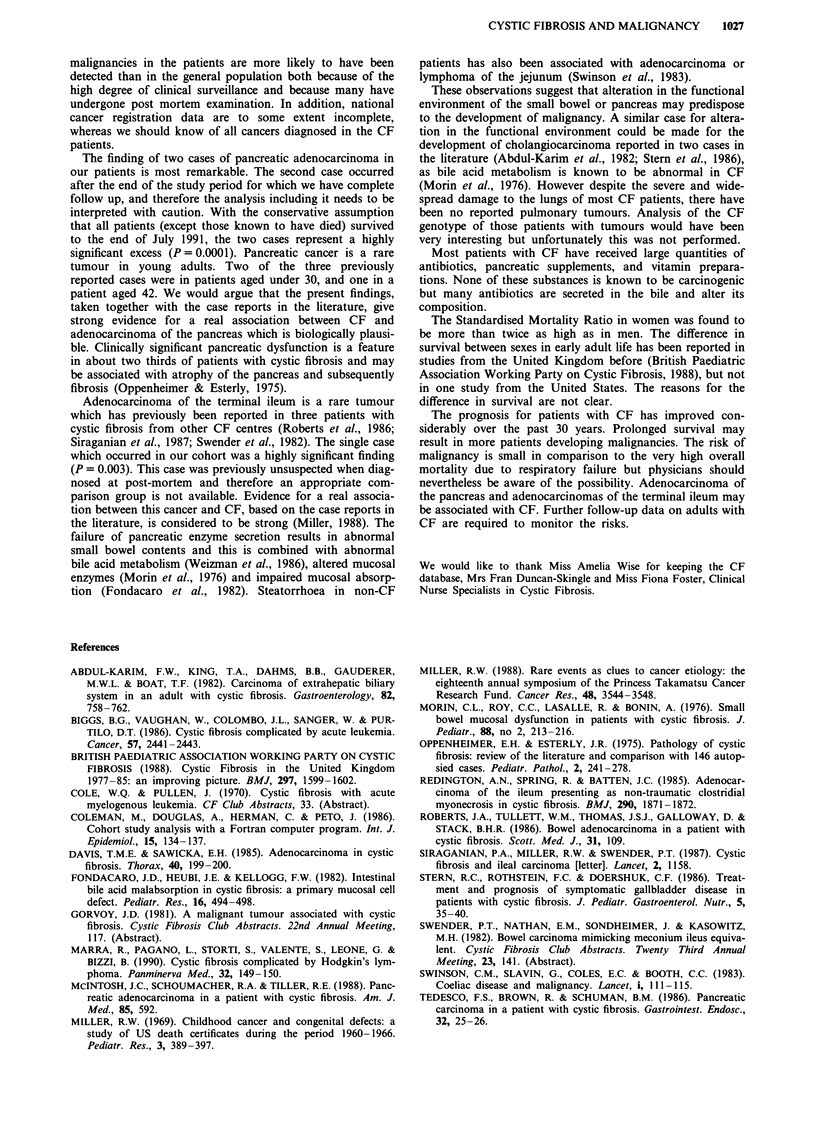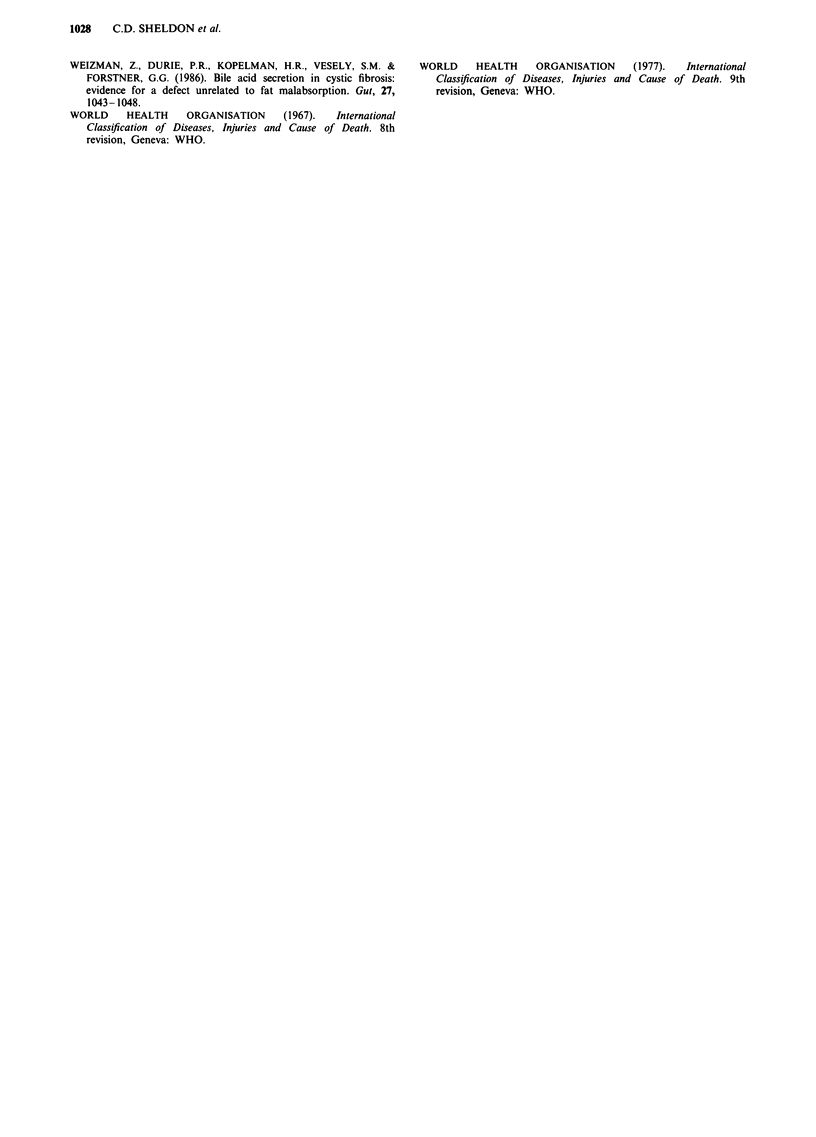# A cohort study of cystic fibrosis and malignancy.

**DOI:** 10.1038/bjc.1993.474

**Published:** 1993-11

**Authors:** C. D. Sheldon, M. E. Hodson, L. M. Carpenter, A. J. Swerdlow

**Affiliations:** Department of Cystic Fibrosis, Royal Brompton National Heart and Lung Hospital (Chelsea), London, UK.

## Abstract

A cohort of 412 patients first attending a cystic fibrosis (CF) clinic between 1961 and 1989 were followed up to 30 June 1989. The number of malignancies observed in the cohort was compared with the number expected based on the age, sex and calendar-year-specific cancer registration rates for England and Wales. Four CF patients were diagnosed as having malignancies before 30 June 1989. The tumours were: adenocarcinoma of the terminal ileum; adenocarcinoma of the pancreas, testicular teratoma, and B-cell lymphoma. This compares with 0.89 malignancies expected on the basis of rates in England and Wales (Standardised Registration Ratio = 452; 95% confidence interval 122-1150, P = 0.03). The single case of adenocarcinoma of the terminal ileum contrasts with less than 0.001 expected (P = 0.003) and that of the pancreas with 0.007 expected (P = 0.01). A further adenocarcinoma of the pancreas was diagnosed 2 years after the end of the study period. The two cases of pancreatic cancer compare with 0.008 expected (P = 0.0001) during the period to mid 1991. On the basis of the present findings and previous case reports in the literature adenocarcinoma of the pancreas and adenocarcinoma of the terminal ileum may be associated with cystic fibrosis.


Br. J. Cancer (1993), 68, 1025-1028 © Macmillan Press Ltd., 1993

### A cohort study of cystic fibrosis and malignancy

C.D. Sheldon[1]; M.E. Hodson[1]; L.M. Carpenter[2] & A.J. Swerdlow[2]

[1]*Department of Cystic Fibrosis, Royal Brompton National Heart and Lung Hospital (Chelsea), Sydney Street, London, SW3;* [2]*Epidemiological Monitoring Unit, Department of Epidemiology and Population Sciences, London School of Hygiene and Tropical Medicine, Keppel Street, London WC1E 7HT, UK.*

**Summary** A cohort of 412 patients first attending a cystic fibrosis (CF) clinic between 1961 and 1989 were followed up to 30 June 1989. The number of malignancies observed in the cohort was compared with the number expected based on the age, sex and calendar-year-specific cancer registration rates for England and Wales. Four CF patients were diagnosed as having malignancies before 30 June 1989. The tumours were: adenocarcinoma of the terminal ileum; adenocarcinoma of the pancreas, testicular teratoma, and B-cell lymphoma. This compares with 0.89 malignancies expected on the basis of rates in England and Wales (Standardised Registration Ratio = 452; 95% confidence interval 122–1150, $P = 0.03$). The single case of adenocarcinoma of the terminal ileum contrasts with less than 0.001 expected ($P = 0.003$) and that of the pancreas with 0.007 expected ($P = 0.01$). A further adenocarcinoma of the pancreas was diagnosed 2 years after the end of the study period. The two cases of pancreatic cancer compare with 0.008 expected ($P = 0.0001$) during the period to mid 1991.

On the basis of the present findings and previous case reports in the literature adenocarcinoma of the pancreas and adenocarcinoma of the terminal ileum may be associated with cystic fibrosis.

Cystic fibrosis (CF) is not usually considered to be associated with malignancy. Until recently the expectation of life in patients with CF was sufficiently short that cancer risk was relatively unimportant. With the introduction of modern treatment methods, however, survival of patients with CF has increased appreciably. There have now been several case reports of malignancy in patients with CF (Abdul-Karim *et al.*, 1982; Biggs *et al.*, 1986; Cole & Pullen, 1970; Davis & Sawicka, 1985; Gorvoy, 1981; Marra *et al.*, 1990; McIntosh *et al.*, 1988; Miller, 1969; Redington *et al.*, 1985; Roberts *et al.*, 1986; Siraganian *et al.*, 1987; Stern *et al.*, 1986; Swender *et al.*, 1982; Tedesco *et al.*, 1986). Determining whether CF patients are at greater risk of cancer is therefore becoming of increasing relevance.

While case reports of CF and malignancy are suggestive of a relationship, these do not allow the association between CF and malignancy to be quantified. In order to assess this, we have examined a cohort of patients with CF who attended The Royal Brompton Hospital between 1961 and 1989. We report here the results of analyses which compared cancer incidence (and all-cause mortality) in the cohort with national rates.

### Patients and methods

The Royal Brompton Hospital is a national referral centre for patients with CF and, since 1961, has had a special interest in older patients with CF. Clinical details of all CF patients first attending the Department of Cystic Fibrosis between 1961 and 1989 were available from hospital records and a computerised data base which is updated regularly. All patients had a diagnosis of CF based on clinical features and a raised sweat sodium ($>70\,\mathrm{mEq\,l^{-1}}$). The majority of patients are seen in outpatients at least every 3 months but patients who are unwell are seen more frequently as necessary. Once seen, patients are generally followed up for life, sometimes in collaboration with a physician at a local hospital. Clinical details of all CF patients under the care of The Royal Brompton Hospital but admitted to other hospitals are obtained by one of the CF clinical nurse specialists or the person responsible for maintaining the computer database.

Person years at risk (pyar) by 5 year age group, sex and calendar year were calculated from the date when the patient was first seen at The Royal Brompton Hospital. Patients ceased contributing pyar on 30th June 1989, or their date of death, or the date last seen alive if either of these were earlier. The number of cancer cases expected in the cohort on the basis of national rates was obtained by multiplying the age, sex, and calendar year-specific pyar by the cancer registration rates recorded for England and Wales in the same age, sex and calendar years. National registration rates for 1971 were used to calculate expected cases before 1971, and 1984 rates used to derive expectations after 1984, since registration rates for these years were not available. A standardised registration ratio (SRR) was calculated as the ratio of the sum of observed to the sum of expected cases, multiplied by 100. Cancer sites were coded to the International Classification of Diseases (ICD), using the revision in force at the time. (World Health Organisation, 1967; World Health Organisation, 1977). Overall mortality in the cohort was analysed in a similar way to give a standardised mortality ratio (SMR), which compared observed deaths to age-, sex-, and calendar year-specific expectations based on national mortality rates. All analyses were performed using the PYRS computer programme. (Coleman *et al.*, 1986).

The statistical significance of SRRs and SMRs, and 95% confidence intervals (CI), were obtained using the Poisson distribution. All tests of significance were two-sided.

### Results

Four patients developed a malignancy during the study period. In addition, one patient was diagnosed as having adenocarcinoma of the pancreas in July 1991. As this case occurred after the period for which detailed follow-up on the whole cohort was available, it was not included in the main part of the analysis. An additional patient within the cohort had a past history of tonsillar lymphoma successfully treated by radiotherapy at age 11 (case not previously reported). His lymphoma was not included in the calculations as the diagnosis had been made before he attended this hospital.

All of the patients developed their malignancy while under routine follow-up and none of the patients was referred to the hospital for specific diagnosis or management of a malignancy.

### *Clinical cases*

*Patient 1* (Davis and Sawicka, 1985) A 23 year old woman diagnosed as having CF at the age of 12 months, presented

Correspondence: C.D. Sheldon.
Received 4 August 1992; and in revised form 6 July 1993.

with vague right sided upper abdominal pain. There was no nausea or vomiting and she described the pain as dull and poorly localised with no relation to eating. Abdominal ultrasound showed a large gall bladder and atrophic pancreas containing a bi-lobular mass in the head. Liver function tests were normal, with no evidence of chronic liver disease. Needle aspiration of the pancreatic mass showed groups of adenocarcinoma cells, confirmed histologically at post-mortem 6 months later.

*Patient 2* (Redington *et al.*, 1985) A 29 year old man had been diagnosed as having CF at the age of 6 months. He developed diabetes mellitus aged 20 and had required admissions for treatment of respiratory symptoms due to infection with *P. aeruginosa*. Liver function tests had been abnormal during the previous two years that he had been attending this hospital and showed a normal bilirubin, markedly elevated alkaline phosphatase (ten times normal) elevated γGT and AST (both about three times normal). He was admitted with a rapidly progressive illness with subcutaneous emphysema and myonecrosis due to *Clostridium septicum* septicaemia. Despite treatment he died within 12 h of admission. At post-mortem an unsuspected moderately well differentiated adenocarcinoma of the terminal ileum was found 1 cm from the ileocaecal valve.

*Patient 3* A 29 year old man, diagnosed as having CF at age 2, was admitted with an exacerbation of respiratory symptoms. He also reported loin pain, dysuria, frequency and a painful swollen right testicle. The right testicle was enlarged and tender; there was no lymphadenopathy or hepatosplenomegaly. Initial haematological and biochemical tests were normal. The serum α-fetoprotein was $15\,\mu g\,l^{-1}$ (normal $<10\,\mu g\,l^{-1}$), serum β-HCG $27\,iu\,l^{-1}$ (normal $<4\,iu\,l^{-1}$) serum CEA $2\,\mu g\,l^{-1}$ (normal $<10\,\mu g\,l^{-1}$). A right orchidectomy showed a teratoma with prominent vascular invasion. Staging investigations showed no evidence of metastatic disease. He was treated with bleomycin, vincristine and cisplatin with no subsequent evidence of tumour recurrence. Sixteen months after the diagnosis of his teratoma he was admitted with a terminal respiratory tract infection. Post-mortem examination showed no evidence of residual tumour.

*Patient 4* A 63 year old man with CF was admitted with an acute exacerbation of his respiratory symptoms. The diagnosis of CF had been made at the age of 50 when he presented with a history of repeated respiratory tract infections and heat prostration in hot climates. He had an elevated sweat sodium which did not suppress with fludrocortisone. Investigations revealed an Hb of $10.9\,g\,l^{-1}$ and WCC of 17.3: the blood film showed occasional prolymphocytes. The ESR was $40\,mm\,hr^{-1}$. Bone marrow examination showed normal appearances apart from reduced erythropoesis and lymphocytic infiltration; approximately 50% of all nucleated cells were lymphocytes. Lymphocytic infiltration compatible with B-cell lymphoma (chronic lymphocytic leukaemia or CLL) was confirmed by cell markers. As he was asymptomatic no treatment was given. He has remained in reasonable health for 4 years apart from respiratory tract infections.

*Patient 5* (case occurring after the study period). A 58 year old man had been diagnosed as having CF at the age of 21. He was treated with pancreatic supplements and infrequent courses of antibiotics for respiratory tract infections. He developed right upper abdominal pain and an abdominal CT scan showed changes suggestive of chronic pancreatitis and a large pseudocyst. He subsequently deteriorated and developed ascites, oedema and a periumbilical mass; biopsy of the mass showed adenorcarcinoma. At post mortem 4 months later there was an 8 cm thick-walled pancreatic pseudocyst, and an irregular 5 cm pancreatic adenocarcinoma obstructing the gastro-oesophageal junction and common bile duct.

*Cohort analysis*

Of the 413 patients seen, there was no follow-up on only one patient. The analyses therefore relate to 412 patients (186 women and 226 men) with cystic fibrosis. These contributed 2708 pyar in total, the average follow-up period being 6.6 years. The majority of patients (315 or 76%) were aged between 15 and 24 years when first seen; only 64 (16%) were first seen at the Royal Brompton Hospital under age 15 and 33 (8%) over age 24. Fifty four (13%) of the patients were first seen before 1971. Of these, the earliest were two patients seen in 1961. A total of 203 deaths (91 in females, 112 in males) were known to have occurred before 30th June 1989. Of the remaining 209 patients, 184 were followed alive to the end of the study period and 25 were lost to follow-up; seven during 1988 or the first half of 1989 and 18 before then. Nine deaths are known to have occurred after the end of the study period and were not included in the mortality analyses.

The 203 deaths observed in the cohort was almost 100 times the 2.1 deaths expected on the basis of national rates (SMR = 9670, 95% Cl 8382–11092). Mortality relative to sex specific national rates was more than twice as high in women (SMR = 16421, 95% Cl 13321–20314) as in men (SMR = 7249, 95% Cl 5950–8695). The four cases of cancer which occurred during follow-up to 30th June 1989 compare with 0.89 malignancies expected on the basis of national rates (SRR = 452, 95% Cl 122–1150, $P = 0.03$). The single case of adenocarcinoma of the terminal ileum contrasts with less than 0.001 expected cancers of the small intestine (ICD 8 and 9: 152) (SRR = 70537, $P = 0.003$) and that of the pancreas with 0.007 expected cancers of the pancreas (ICD 8 and 9: 157) (SRR = 14379, $P = 0.01$).

In order to assess the statistical significance of the second case of pancreatic cancer, additional analyses were performed assuming that all patients alive at the end of June 1989 survived to the end of July 1991, except that the nine patients known to have died during this period were censored at their date of death. On these conservative assumptions, the two cases of pancreatic cancer compare with 0.008 expected cases (SRR = 24448, $P = 0.0001$).

### Discussion

Malignancies occurring in cystic fibrosis are rare and we can find only 19 cases reported in the literature; six cases of leukaemia (Miller, 1969; Cole & Pullen, 1970; Biggs *et al.*, 1986), one case of Hodgkins disease (Marra *et al.*, 1990), four cases of adenocarcinoma of the terminal ileum (Roberts *et al.*, 1986; Siraganian *et al.*, 1987; Swender *et al.*, 1982; Redington *et al.*, 1985), two nephroblastomas (Wilms' tumour) (Miller, 1969), three cases of adenocarcinoma of the pancreas (Tedesco *et al.*, 1986; Davis & Sawicka, 1985; McIntosh *et al.*, 1988), a case of retinoblastoma (Gorvoy, 1981) and two cholangiocarcinomas (Abdul-Karim *et al.*, 1982; Stern *et al.*, 1986). While suggestive of a relationship between CF and malignancy, these case reports do not allow the strength of the association to be quantified. This study is the first report of a cohort study of malignancy in patients with CF.

The more than 4-fold excess of malignant tumours observed in our cohort provides firmer evidence than previously available for an association between CF and the development of malignancy. This could be due to a direct effect associated with the CF genetic defect or it may be due to a secondary effect in an organ affected by the CF disease process. It should be noted however, that patients included in this study were all under follow up for CF and attended this hospital regularly. As The Royal Brompton Hospital is a tertiary referral hospital our cohort represents a relatively selected population. None of the patients was referred to us because of symptoms associated with the cancer: four of the five cases were diagnosed more than 10 years after first registration at the hospital and one case, two and a half years after registration. As most patients are followed up for life, malignancies in the patients are more likely to have been detected than in the general population both because of the high degree of clinical surveillance and because many have undergone post mortem examination. In addition, national cancer registration data are to some extent incomplete, whereas we should know of all cancers diagnosed in the CF patients.

The finding of two cases of pancreatic adenocarcinoma in our patients is most remarkable. The second case occurred after the end of the study period for which we have complete follow up, and therefore the analysis including it needs to be interpreted with caution. With the conservative assumption that all patients (except those known to have died) survived to the end of July 1991, the two cases represent a highly significant excess ($P = 0.0001$). Pancreatic cancer is a rare tumour in young adults. Two of the three previously reported cases were in patients aged under 30, and one in a patient aged 42. We would argue that the present findings, taken together with the case reports in the literature, give strong evidence for a real association between CF and adenocarcinoma of the pancreas which is biologically plausible. Clinically significant pancreatic dysfunction is a feature in about two thirds of patients with cystic fibrosis and may be associated with atrophy of the pancreas and subsequently fibrosis (Oppenheimer & Esterly, 1975).

Adenocarcinoma of the terminal ileum is a rare tumour which has previously been reported in three patients with cystic fibrosis from other CF centres (Roberts *et al.*, 1986; Siraganian *et al.*, 1987; Swender *et al.*, 1982). The single case which occurred in our cohort was a highly significant finding ($P = 0.003$). This case was previously unsuspected when diagnosed at post-mortem and therefore an appropriate comparison group is not available. Evidence for a real association between this cancer and CF, based on the case reports in the literature, is considered to be strong (Miller, 1988). The failure of pancreatic enzyme secretion results in abnormal small bowel contents and this is combined with abnormal bile acid metabolism (Weizman *et al.*, 1986), altered mucosal enzymes (Morin *et al.*, 1976) and impaired mucosal absorption (Fondacaro *et al.*, 1982). Steatorrhoea in non-CF patients has also been associated with adenocarcinoma or lymphoma of the jejunum (Swinson *et al.*, 1983).

These observations suggest that alteration in the functional environment of the small bowel or pancreas may predispose to the development of malignancy. A similar case for alteration in the functional environment could be made for the development of cholangiocarcinoma reported in two cases in the literature (Abdul-Karim *et al.*, 1982; Stern *et al.*, 1986), as bile acid metabolism is known to be abnormal in CF (Morin *et al.*, 1976). However despite the severe and widespread damage to the lungs of most CF patients, there have been no reported pulmonary tumours. Analysis of the CF genotype of those patients with tumours would have been very interesting but unfortunately this was not performed.

Most patients with CF have received large quantities of antibiotics, pancreatic supplements, and vitamin preparations. None of these substances is known to be carcinogenic but many antibiotics are secreted in the bile and alter its composition.

The Standardised Mortality Ratio in women was found to be more than twice as high as in men. The difference in survival between sexes in early adult life has been reported in studies from the United Kingdom before (British Paediatric Association Working Party on Cystic Fibrosis, 1988), but not in one study from the United States. The reasons for the difference in survival are not clear.

The prognosis for patients with CF has improved considerably over the past 30 years. Prolonged survival may result in more patients developing malignancies. The risk of malignancy is small in comparison to the very high overall mortality due to respiratory failure but physicians should nevertheless be aware of the possibility. Adenocarcinoma of the pancreas and adenocarcinomas of the terminal ileum may be associated with CF. Further follow-up data on adults with CF are required to monitor the risks.

We would like to thank Miss Amelia Wise for keeping the CF database, Mrs Fran Duncan-Skingle and Miss Fiona Foster, Clinical Nurse Specialists in Cystic Fibrosis.

### References

ABDUL-KARIM, F.W., KING, T.A., DAHMS, B.B., GAUDERER, M.W.L. & BOAT, T.F. (1982). Carcinoma of extrahepatic biliary system in an adult with cystic fibrosis. *Gastroenterology*, **82**, 758–762.

BIGGS, B.G., VAUGHAN, W., COLOMBO, J.L., SANGER, W. & PURTILO, D.T. (1986). Cystic fibrosis complicated by acute leukemia. *Cancer*, **57**, 2441–2443.

BRITISH PAEDIATRIC ASSOCIATION WORKING PARTY ON CYSTIC FIBROSIS (1988). Cystic Fibrosis in the United Kingdom 1977–85: an improving picture. *BMJ*, **297**, 1599–1602.

COLE, W.Q. & PULLEN, J. (1970). Cystic fibrosis with acute myelogenous leukemia. *CF Club Abstracts*, 33. (Abstract).

COLEMAN, M., DOUGLAS, A., HERMAN, C. & PETO, J. (1986). Cohort study analysis with a Fortran computer program. *Int. J. Epidemiol.*, **15**, 134–137.

DAVIS, T.M.E. & SAWICKA, E.H. (1985). Adenocarcinoma in cystic fibrosis. *Thorax*, **40**, 199–200.

FONDACARO, J.D., HEUBI, J.E. & KELLOGG, F.W. (1982). Intestinal bile acid malabsorption in cystic fibrosis: a primary mucosal cell defect. *Pediatr. Res.*, **16**, 494–498.

GORVOY, J.D. (1981). A malignant tumour associated with cystic fibrosis. *Cystic Fibrosis Club Abstracts. 22nd Annual Meeting*, 117. (Abstract).

MARRA, R., PAGANO, L., STORTI, S., VALENTE, S., LEONE, G. & BIZZI, B. (1990). Cystic fibrosis complicated by Hodgkin's lymphoma. *Panminerva Med.*, **32**, 149–150.

MCINTOSH, J.C., SCHOUMACHER, R.A. & TILLER, R.E. (1988). Pancreatic adenocarcinoma in a patient with cystic fibrosis. *Am. J. Med.*, **85**, 592.

MILLER, R.W. (1969). Childhood cancer and congenital defects: a study of US death certificates during the period 1960–1966. *Pediatr. Res.*, **3**, 389–397.

MILLER, R.W. (1988). Rare events as clues to cancer etiology: the eighteenth annual symposium of the Princess Takamatsu Cancer Research Fund. *Cancer Res.*, **48**, 3544–3548.

MORIN, C.L., ROY, C.C., LASALLE, R. & BONIN, A. (1976). Small bowel mucosal dysfunction in patients with cystic fibrosis. *J. Pediatr.*, **88**, no 2, 213–216.

OPPENHEIMER, E.H. & ESTERLY, J.R. (1975). Pathology of cystic fibrosis: review of the literature and comparison with 146 autopsied cases. *Pediatr. Pathol.*, **2**, 241–278.

REDINGTON, A.N., SPRING, R. & BATTEN, J.C. (1985). Adenocarcinoma of the ileum presenting as non-traumatic clostridial myonecrosis in cystic fibrosis. *BMJ*, **290**, 1871–1872.

ROBERTS, J.A., TULLETT, W.M., THOMAS, J.S.J., GALLOWAY, D. & STACK, B.H.R. (1986). Bowel adenocarcinoma in a patient with cystic fibrosis. *Scott. Med. J.*, **31**, 109.

SIRAGANIAN, P.A., MILLER, R.W. & SWENDER, P.T. (1987). Cystic fibrosis and ileal carcinoma [letter]. *Lancet*, **2**, 1158.

STERN, R.C., ROTHSTEIN, F.C. & DOERSHUK, C.F. (1986). Treatment and prognosis of symptomatic gallbladder disease in patients with cystic fibrosis. *J. Pediatr. Gastroenterol. Nutr.*, **5**, 35–40.

SWENDER, P.T., NATHAN, E.M., SONDHEIMER, J. & KASOWITZ, M.H. (1982). Bowel carcinoma mimicking meconium ileus equivalent. *Cystic Fibrosis Club Abstracts. Twenty Third Annual Meeting*, **23**, 141. (Abstract).

SWINSON, C.M., SLAVIN, G., COLES, E.C. & BOOTH, C.C. (1983). Coeliac disease and malignancy. *Lancet*, **i**, 111–115.

TEDESCO, F.S., BROWN, R. & SCHUMAN, B.M. (1986). Pancreatic carcinoma in a patient with cystic fibrosis. *Gastrointest. Endosc.*, **32**, 25–26.

WEIZMAN, Z., DURIE, P.R., KOPELMAN, H.R., VESELY, S.M. & FORSTNER, G.G. (1986). Bile acid secretion in cystic fibrosis: evidence for a defect unrelated to fat malabsorption. *Gut*, **27**, 1043–1048.

WORLD HEALTH ORGANISATION (1967). *International Classification of Diseases, Injuries and Cause of Death*. 8th revision, Geneva: WHO.

WORLD HEALTH ORGANISATION (1977). *International Classification of Diseases, Injuries and Cause of Death*. 9th revision, Geneva: WHO.